# Supplementary material for: Selective signatures in composite MONTANA TROPICAL beef cattle reveal potential genomic regions for tropical adaptation
Source: PLoS One. 2024 Apr 25;19(4):e0301937. doi: 10.1371/journal.pone.0301937 (PMC11045132; doi:10.1371/journal.pone.0301937)
Supplement: S1 Table — Standard error (SE), mean (Mb), standard deviation (SD), min (Mb), max (Mb), SROH (mean length of genome covered by ROH Mb), NROH (mean number of ROH), LROH (mean length of ROH in Mb) and FROH (inbreeding coefficient). (PDF) [file pone.0301937.s001.pdf]

**S1. Statistical Homozygosity Runs (ROH) per animal**

| Total Sample | Mean | *SE* | *SD* | Min | Max |
|---|---|---|---|---|---|
| **SROH** | 61,21 | 1,10 | 62,89 | 0 | 845,53 |
| **NROH** | 7,48 | 0,08 | 4,97 | 0 | 39 |
| **LROH** | 8,18 | 0,05 | 7,81 | 1,01 | 109,11 |
| **FROH** | 0,02 | 0,0004 | 0,02 | 0 | 0,31 |

Standard error (SE), mean (Mb), standard deviation (SD), min (Mb), max (Mb), SROH (mean length of genome covered by ROH Mb), NROH (mean number of ROH), LROH (mean length of ROH in Mb) and FROH (inbreeding coefficient)
