# Supplementary material for: Selective signatures in composite MONTANA TROPICAL beef cattle reveal potential genomic regions for tropical adaptation
Source: PLoS One. 2024 Apr 25;19(4):e0301937. doi: 10.1371/journal.pone.0301937 (PMC11045132; doi:10.1371/journal.pone.0301937)
Supplement: S2 Table — (PDF) [file pone.0301937.s002.pdf]

Approach	Generation	Mb start	Mb end	Size
ROH	All	35.39	40.24	4.84
ROH	2.5<ECG<4.5	35.39	39.08	3.68
ROH	ECG>4.5	35.20	39.72	4.5
iHS	All	21.20	23.20	2.00
iHS	All	24.94	35.09	10.15
iHS	All	35.30	40.32	5.02
iHS	ECG<2.5	21.19	23.21	2.02
iHS	ECG<2.5	26.35	34.36	8.00
iHS	ECG<2.5	35.30	38.96	3.66
iHS	2.5<ECG<4.5	21.20	23.20	2.00
iHS	2.5<ECG<4.5	24.49	34.45	9.95
iHS	2.5<ECG<4.5	35.30	40.32	5.02
iHS	2.5<ECG<4.5	41.11	43.11	2.00
iHS	ECG>4.5	26.73	29.37	2.63
iHS	ECG>4.5	41.11	43.13	2.01
iHS	ECG>4.5	44.89	46.89	2.00
iHS	ECG>4.5	47.52	49.52	2.00

S2 Table. All regions that was found by iHS and ROH, through different Equivalent complete generations (ECG) in the consensus region of BTAU 20.

S1 File. Estimated iHS SNP in the MONTANA TROPICAL®
