# Supplementary figures and images for: Selective signatures in composite MONTANA TROPICAL beef cattle reveal potential genomic regions for tropical adaptation
Source: PLoS One. 2024 Apr 25;19(4):e0301937. doi: 10.1371/journal.pone.0301937 (PMC11045132; doi:10.1371/journal.pone.0301937)

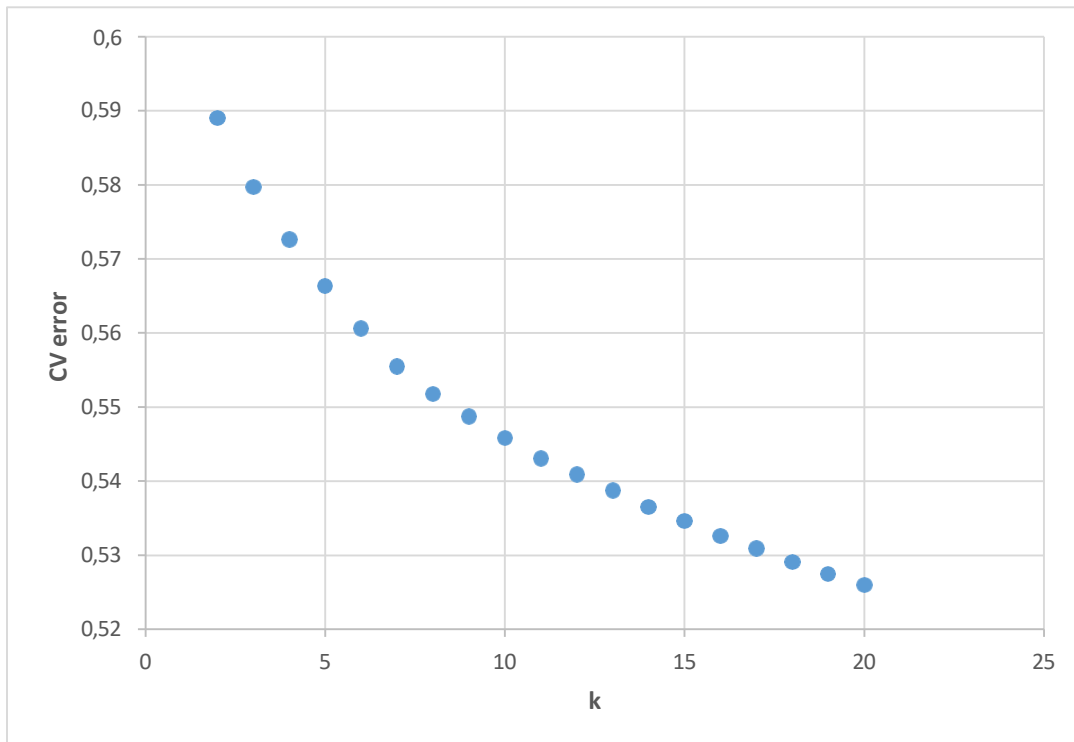

**S3 File. CV error of different value of K.**

Supplement: S3 File — (PDF) [file pone.0301937.s005.pdf]

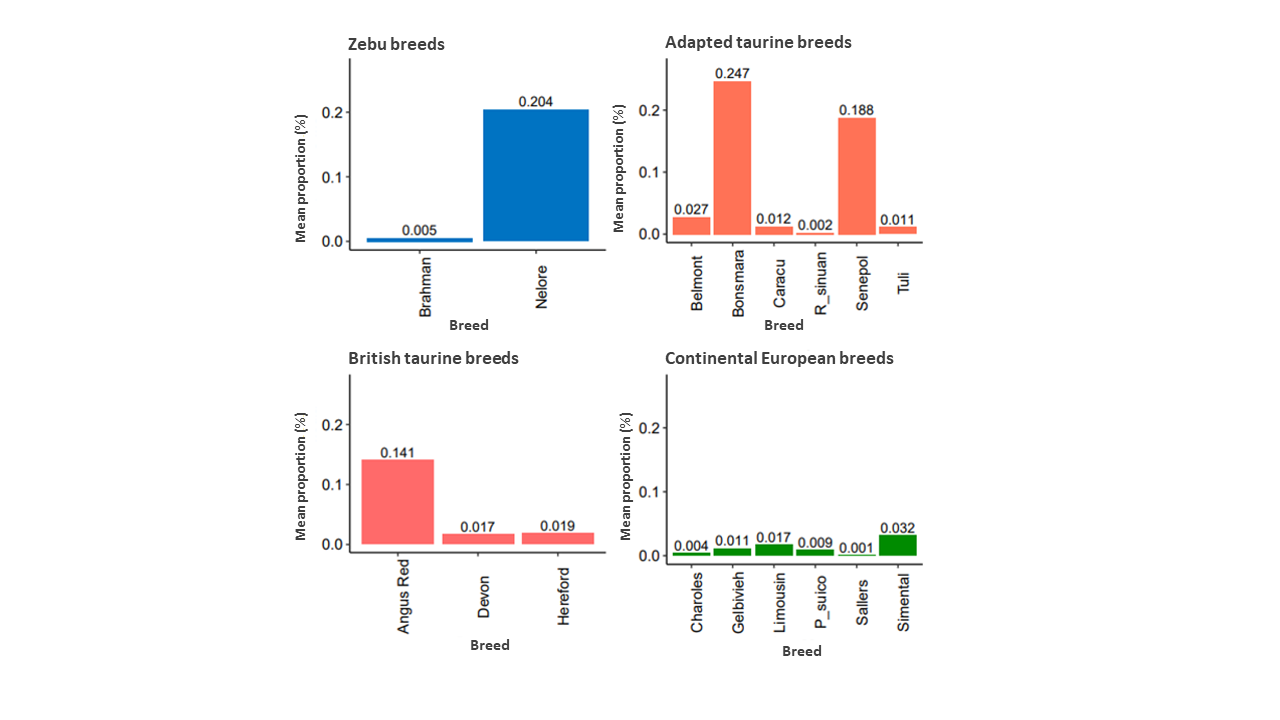

Supplement: S1 Fig — (TIF) [file pone.0301937.s007.tif]

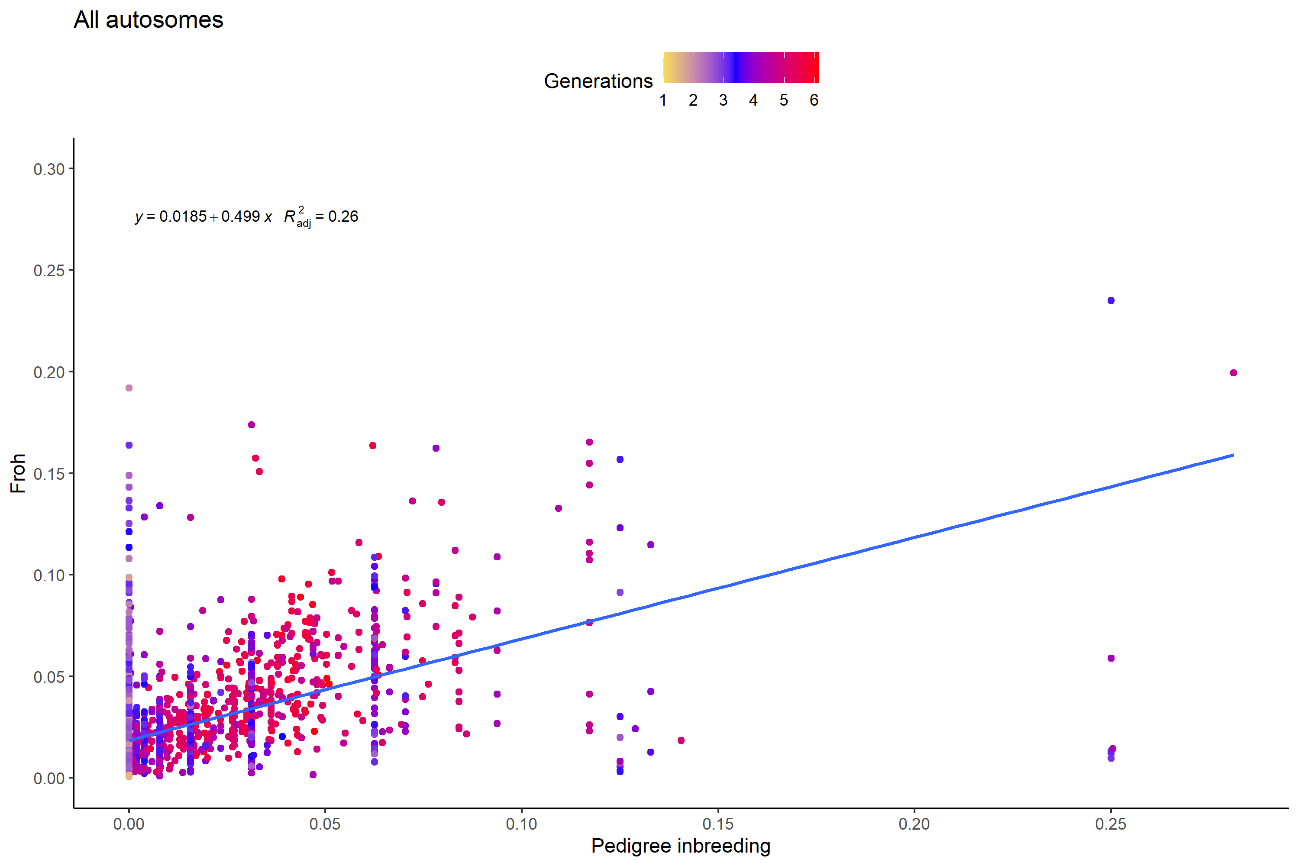

Supplement: S2 Fig — (TIF) [file pone.0301937.s008.tif]

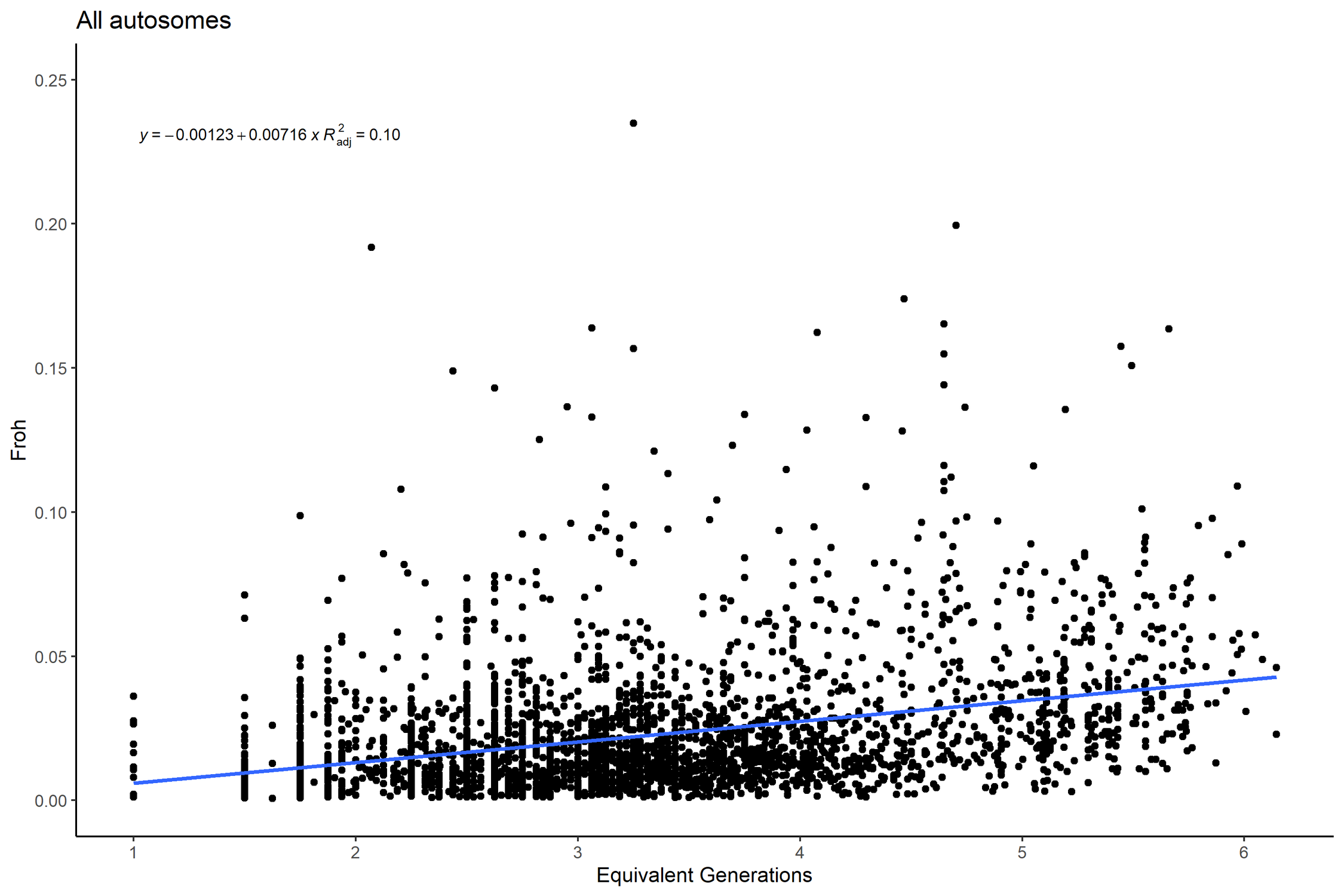

Supplement: S3 Fig — (TIF) [file pone.0301937.s009.tif]

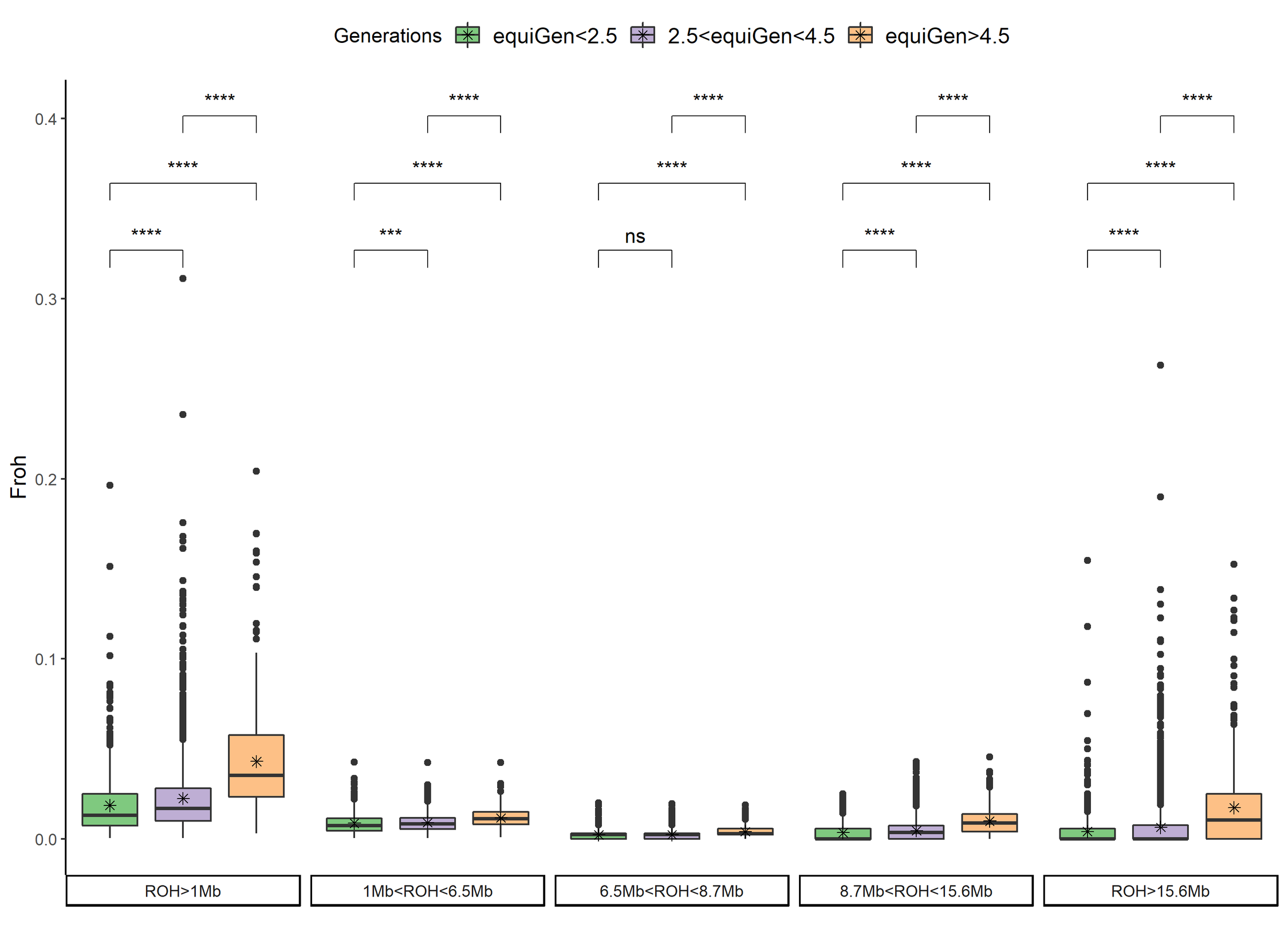

Supplement: S4 Fig — The t-test comparison results are shown at the top (ns: not significant; *p< 0.05; **p< 0.01; ***p< 0.001; ****p< 0.0001). (TIF) [file pone.0301937.s010.tif]

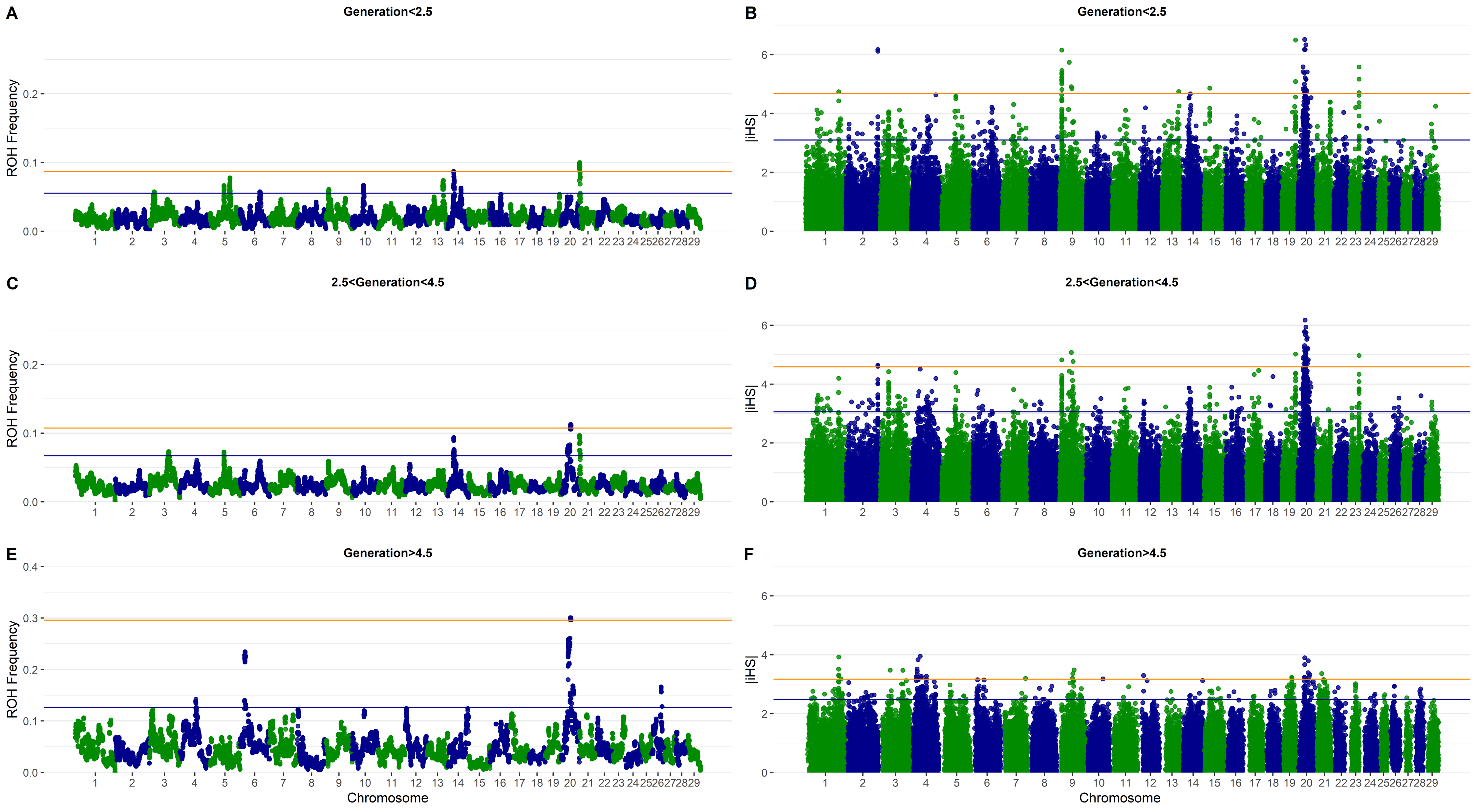

Supplement: S5 Fig — A, C, E Manhattan plot of incidence of each SNP in the ROH across individuals through different ECGs. The blue line represents the top 0.1% threshold Manhattan plot of incidence of each SNP in the ROH across individuals. The orange line represents the threshold levels of top 0.1%. of Genome-wide distribution of selection signatures detected by iHS B, D, F through different ECGs. (TIF) [file pone.0301937.s011.tif]

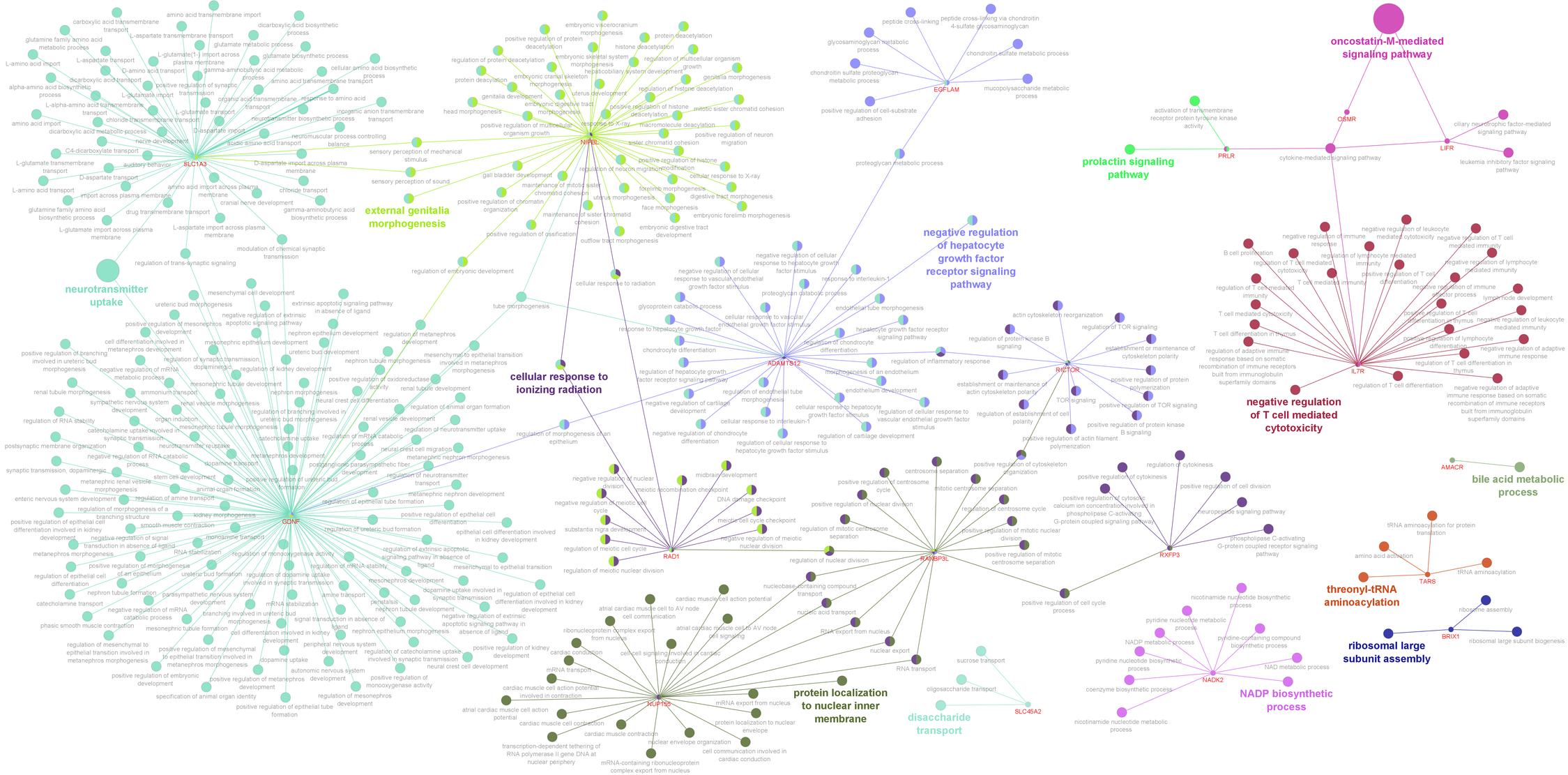

Supplement: S6 Fig — (TIF) [file pone.0301937.s012.tif]
