# Supplementary material for: Selective signatures in composite MONTANA TROPICAL beef cattle reveal potential genomic regions for tropical adaptation
Source: PLoS One. 2024 Apr 25;19(4):e0301937. doi: 10.1371/journal.pone.0301937 (PMC11045132; doi:10.1371/journal.pone.0301937)
Supplement: Attachment — Submitted filename: Response to Reviewers.pdf [file pone.0301937.s013.pdf]

We appreciate the reviewer's and editor's considerations, and we address all questions. Hereinafter, we indicate the changes made point by point in red.

Editor:

1.Please ensure that your manuscript meets PLOS ONE's style requirements, including those for file naming. The PLOS ONE style templates…

We used both templates available at https://journals.plos.org/plosone/s/submission-guidelines to prepare our manuscript for the submission . (https://journals.plos.org/plosone/s/file?id=3fac/PLOS%20Affiliations%20Formatting%20Guidelines.pdf https://journals.plos.org/plosone/s/file?id=9cba/PLOS%20Manuscript%20Body%20Formatting%20Guidelines.pdf)

2.Please note that funding information should not appear in any section or other areas of your manuscript. We will only publish funding information present in the Funding Statement section of the online submission form. Please remove any funding-related text from the manuscript.

We removed all the founding information that was in the Manuscript and indicate the change in the cover letter as requested.

3.Thank you for stating the following financial disclosure: "CNPq"

Please state what role the funders took in the study. If the funders had no role, please state: "The funders had no role in study design, data collection and analysis, decision to publish, or preparation of the manuscript." If this statement is not correct you must amend it as needed. Please include this amended Role of Funder statement in your cover letter; we will change the online submission form on your behalf.

The Financial Disclosure: Instituto Federal de Educação, Ciência e Tecnologia Goiano (IF Goiano) and National Council for Scientific and Technological Development (CNPq). The funders had no role in study design, data collection and analysis, decision to publish, or preparation of the manuscript only in the financial support and the infrastructure."

4. We note that you have stated that you will provide repository information for your data at acceptance. Should your manuscript be accepted for publication, we will hold it until you provide the relevant accession numbers or DOIs necessary to access your data. If you wish to make changes to your Data Availability statement, please describe these changes in your cover letter and we will update your Data Availability statement to reflect the information you provide.

Sorry the statement it was a mistake our final data availability statement it is:

“Data Availability - No - some restrictions will apply”

“Data cannot be shared publicly because of is under control of the breeders association. Data only will be available from the Montana Tropical breeder association (contato@montana.org.br) for researchers who ask and meet their criteria for access to Confidencial data.”

Addition2: we note that the link provided leads to a general homepage. At this time, please provide contact information where fellow researchers can direct data inquiries.

We provided the direct contact information as requested in the above statement and in the cover letter.

Reviewer #1:

Please consider carefully all comments below:

- I want to thank you for placing figures and tables within the text! (and not at the end of it)
- but please add line numbers!!
- English: overall not too bad, but check some typos and especially the many poorly constructed sentences
- Methods must be described more precisely, with less ambiguity and providing as much as possible the exact calculations that you used

We added line numbers and performed a carefully English check. Moreover, we revised the description of methods used.

Background

P10: changing climate scenario – done

P10: composed of – done

P11: arose - done

P11: succeeded - done

P11: do you have a reference/link for the registered Montana Tropical cattle? If it's a trademark/brand, please always capitalize it (including in the title) – we added a citation of the Association website in line 52

P11: composite breed – done

P11: based on THE crossbreeding OF four – done.

P11: "has growth traits as 70% of their selection index" please rephrase --> growth traits account for 70% of their selection index – done.
P12: I guess you refer to pedigree errors (you can find something on this in the literature, e.g. Leroy et al. 2012 Animal Genetics) – done, It was a missing word before the errors.
P12: history of population, not of pop structure – done.
P12: rephrase to --> The population frequency of ROH can be used as a tool to identify genomic regions under selection (i.e. signatures of selection) - done
P12: Another method to detect signals of recent selection is the integrated haplotype score ... – done
P12: are you sure that iHS only detects recent seelction?
According to the Voight et al. (2006), iHS method detect more recent signal of selection compared to other methods available. To avoid any misinterpretation, we removed “recent” from the statement. L:84
Voight, B. F., Kudaravalli, S., Wen, X., & Pritchard, J. K. (2006). A map of recent positive selection in the human genome. *PLoS biology, 4*(3), e72.

Methods

P27: "At the end, it was used 3215 genotypes (HD GGP 50K Neogen, genome reference URS UCD 1.2) for the further analyses presented here.": wrongly written (it was used?), please rewrite – done

P27: before performing – done

P28: signatures of selection – done

P28: , according – done

P28: what is the completeness value? How is it calculated?
The completeness value is an index of pedigree completeness, which is the harmonic mean of the pedigree completeness of the parents. The relevant parameter to identify individuals with insufficient pedigree information to estimate inbreeding, however, is the PCI. This is because inbreeding can be detected only if both maternal and paternal ancestries are known. The harmonic mean ensures that the less complete paternal pedigree is weighted more heavily, so the PCI equals zero when either parent is unknown. Inbreeding coefficients can be valid despite small PCIs if the most recent founders were indeed unrelated, e.g. because they were from other breeds. L:373

MacCluer JW, Boyce AJ, Dyke B, Weitkamp LR, Pfenning DW, Parsons CJ. Inbreeding and pedigree structure in Standardbred horses. J Hered. 1983; 1;74(6):394–9. doi:10.1093/oxfordjournals.jhered.a109824.

P28: breed proportion – done

P28: what is the purpose of the ECG?

In the bovine mating system normally occurs the overlapping of generations. As example, a 7th generation dam can be mated with a 4th generation sire. The ECG formula accounts for this overlapping and provide a result that demonstrate the relationship of the animal with the initial crossbreeding for composite formation. In our case, we considered the animal born from a crossbreeding between founder's breed that meet Montana requirements as a 1st generation. Therefore, we used ECG as indicator of how many generations each animal has inside the composite breed.

P28: "standardized variance relationship matrix"? What is this? Usually, PCA is performed on the matrix of genotypes, or on the matrix of genetic relationships calculated from the genotypes (in this latter case, we talk more precisely of PCoA: principal coordinates analysis)

We performed the PCA analysis using the genetic relationship matrix. This analysis as done using PLINK default conditions. PLINK manual (https://www.cog-genomics.org/plink/1.9/strat#pca) indicates that the command "--pca" calculate the principal components of the variance-standardized relationship matrix". We only changed the number of retained PC according to the Kaiser's rule. L: 385

P28: how did you choose the number 10? (I guess that you used the first 10 PCs ordered by decreasing eigenvalues or transformations thereof)

We followed Kaiser's rule, thus we retained the number of components whose eigenvalues were greater than 1. We added this information in the text. L:388

Kaiser, H. F. (1991). Coefficient alpha for a principal component and the Kaiser-Guttman rule. Psychological reports, 68(3), 855-858.

P29: to determine K, besides the CV error you can look also at the number of iterations needed to converge (you can see an example and explanation in rice populations from Biscarini et al. 2016, Plos One)

We appreciate your suggestion. We checked the number of iterations to convergence in each run, however we still not been able to determine the best K. The minimal number of iterations was 23.3 (K=2) and the maximum was 50.7 with K = 13, then the number of iterations decreased, resulting in 40.1 iterations to converge with K=20. As mentioned by Biscarini et al. 2016 and Alexander et al. 2019, we expected that the number of iterations needed rapidly increases when the data start to support poorly the tested number of clusters (K).

P30: density of ONE SNP per 100 kb – done

P30: LROH, LAUTO: ROH and AUTO should be suffixes here – done

P30: "The incidence of common ROH was transformed to generation class frequency by

dividing by the number of animals from each generation class in the analysis." This is not clear, please explain better or add the calculations that you performed (preferred option) L:429

The animals were grouped according to generation classes - all generations, ECG<2.5, 2.5<ECG<4.5 and ECG>4.5. The incidence of common ROH was calculated to each one of these groups.

P30: normality test of what? Which test? Shapiro-wilk test of normality of the ROH results. L:430

P30: top 0.1% of observations in terms of what? Not clear

We calculate the threshold as top 0.1% of ROH frequency based on a gaussian distribution. In termers of the ROH frequency, this can be observed in the Manhattan plot whose the orange line represents the threshold levels of top 0.1 %.

P31: what was the cM --> Mb conversion rate that you used? 1cM = 1Mb?

We calculated the average of the results obtained by Arias et al. (2009) and Weng et al. (2014) to obtain the conversion rate as stated in line 444. Both studies performed a detailed analysis of the bovine recombination map.

P31: GO analysis - done

Reviewer #2:

1.Some information (for example, the description of calculated parameters, and used software) in the Results section should be moved to Materials and Methods.

We removed most of these descriptions, however some of them we believed that it is necessary to explain the basic of what we have done to make the results understandable, as the material and methods is presented at the end of the paper.

2. The captions for Figure 2 are hardly readable. – We remade the plot increasing the size of the captions to get a more readable figure.

3.The authors stated that FROH showed a positive correlation with inbreeding coefficient based on pedigree. I recommend providing these calculations in the Table (or as Supplementary Table). Was the correlation significant?

Yes, it was significant. We added these results as Supplementary Table.

4. Figure 5 should be provided in a better quality.

We tried our best to provide a better quality image.

5.The photograph of Montana Tropical cattle will attract attention of potential readers, who are not familiar with this breed – We added a link to Breeder's Association website and a photo of the animals in the text.
