# Supplementary material for: Selective signatures in composite MONTANA TROPICAL beef cattle reveal potential genomic regions for tropical adaptation
Source: PLoS One. 2024 Apr 25;19(4):e0301937. doi: 10.1371/journal.pone.0301937 (PMC11045132; doi:10.1371/journal.pone.0301937)
Supplement: Attachment — Submitted filename: Response_to_rewiers.docx [file pone.0301937.s014.docx]

We appreciate the reviewer’s considerations, and we address all questions. Hereinafter, we indicate the changes made point by point in red.

Review Comments to the Author

Reviewer #1:

Abstract
--------
- "Genomic regions related to tropical adaptability are OF paramount IMPORTANCE for animal breeding nowadays, especially ... " – Done

- "Moreover, the genomic architecture of these traits MAY BE very RELEVANT FOR breeding programs." Why do you think that the genomic architecture of these traits is relevant for breeding programmes: pelase explain (also in the text of the Abstract)

To know the genetic architecture of this regions is important for geneticists and breeding programs so they can make more informed decisions when selecting animals with objectives related to tropical adaptation trough hybridization for example.

We also complement the text in the abstract.

- " ... were used to: i) characterize the population structure: ii) identify signatures of selection with complementary approaches, i.e. Integrated Haplotype Score (iHS) and Runs of Homozygosity (ROH); and iii) understand genes and traits related to selected region" – Done


Methods
-------
- I appreciated your response to my comments on "completeness value" and "ECG": please add part of those replies to the text of the manuscript

-We added part of the explanations in the text, for completeness of the pedigree L374 – L377 and for ECG L383 – L389.

Reviewer #2:

The authors addressed comment raised during review process.
Figure 1 is missing in the revised manuscript. Please check it before the publication. - We appreciate your comment, but in our last reviewed manuscript, we choose the option to attach the figures separately in the Editorial Manager page.

All gene names should be in italics. Check it. - Done
