# Supplementary material for: Selective signatures in composite MONTANA TROPICAL beef cattle reveal potential genomic regions for tropical adaptation
Source: PLoS One. 2024 Apr 25;19(4):e0301937. doi: 10.1371/journal.pone.0301937 (PMC11045132; doi:10.1371/journal.pone.0301937)
Supplement: Attachment — Submitted filename: ResponsetoReviwersV3.docx [file pone.0301937.s015.docx]

We appreciate the reviewer's considerations. We have addressed the changes and also conducted an English review of the document. We have indicated the changes made point by point in red.

Reviewer #1:

 Please carefully check the English. There are some sentences which are poorly written: I provide a couple of examples below, but there are more.

**L392-393**: "With the harmonic mean the less complete paternal pedigree is weighted more heavily, i.e. when both parents are unknow the PCI is equal 0" This sentence is not well written: try something like "The harmonic means place a higher weight on less complete paternal pedigrees, i.e. ... "

Done. We appreciate the suggestion.

**L399-400**: "In the bovine mating system normally occurs the overlapping of generations": again, not well written. Try "In cattle, normally animals mate across overlapping generations"

Done.
